# The complete plastome of *Hyacinthoides non-scripta* (L.) Chouard ex Rothm. (Asparagaceae)

**DOI:** 10.1080/23802359.2020.1720543

**Published:** 2020-02-03

**Authors:** George J. L. Garnett, Kálmán Könyves, Jordan Bilsborrow, John David, Alastair Culham

**Affiliations:** a Herbarium, School of Biological Sciences, University of Reading, Reading, United Kingdom;; b Royal Horticultural Society Garden Wisley, Woking, United Kingdom

**Keywords:** Hyacinthoides, Asparagaceae, plastome, *infA*

## Abstract

The whole plastome sequence of *Hyacinthoides non-scripta*, was assembled and annotated in this study. This is the first complete plastid genome for the genus *Hyacinthoides*. The plastome is 155,035 bp long and consists of a large single-copy (LSC) region spanning 83,947 bp, a small single-copy (SSC) region spanning 18,496 bp, and two inverted repeat (IR) regions, each of which is 26,296 bp in length. There are 132 genes annotated in the plastome, of which the protein-coding gene *infA* has pseudogenized.

*Hyacinthoides* Heist. ex Fabr. is a small genus of bulbous plants in Asparagaceae subfamily Scilloideae. *Hyacinthoides non-scripta*, the English bluebell, is one of the most widespread species in the genus, occurring across western Europe (Grundmann et al. 2010). The largest populations of this species exist in the British Isles, where it is a well known spring-flowering plant (Kohn et al. 2009). Delimitation of genera within Scilloideae is in flux (Speta 1998; POWO 2020). A comprehensive phylogenetic framework based on next-generation sequencing data will help in understanding the generic limits within the subfamily. Here we report the complete plastome of *H. non-scripta* as the first part of a wider project.

Leaf material was collected from a single *H. non-scripta* plant growing naturally on the University of Reading, Whiteknights campus (51°26′16′′N, 0°56′26′′W). A herbarium voucher was deposited at University of Reading Herbarium (RNG, G. Garnett 1). Total genomic DNA was extracted using a Qiagen DNeasy Plant Mini Kit. Library development and Illumina HiSeq 150 bp PE sequencing were completed by the Novogene Company Limited (Beijing, China). The plastome was assembled with Fast-Plast v1.2.8 (McKain and Wilson 2017) and NovoPlasty v3.7.0 (Dierckxsens et al. 2017). Reads were trimmed to remove NEB-PE adapter sequences. The Bowtie reference index was built with Asparagales plastomes included in Fast-Plast. Using NovoPlasty, a *matK* sequence of *H. non-scripta* (JX090371; Voucher specimen: Fay, M.F. MFF108 K) was used as the starting seed. All other parameters were unchanged. A large single-copy (LSC), small single-copy (SSC), and two inverted repeat (IR) regions were identified in the Fast-Plast and NovoPlasty assemblies and the closed circular plastome was assembled by hand using Geneious Prime 2020.0.4 (https://www.geneious.com). Junctions of the single-copy and inverted repeat regions were confirmed following Könyves et al. (2019).

The mean coverage of the finished assembly is 74×. The complete plastome was annotated from *Barnardia japonica* (KX822775; Voucher specimen: Hana140807-3) using Geneious Prime 2020.0.4 and corrected by comparing it with other published annotations. The *H. non-scripta* plastome was aligned to 18 published plastomes across Asparagaceae and two outgroup sequences using MAFFT v7.450 (Katoh and Standley 2013). A maximum-likelihood estimate of phylogeny was conducted with RAxML v8.2.11 (Stamatakis 2014) within Geneious Prime 2020.0.4 using the model GTR + I + G and 1000 bootstrap replicates.

The plastome sequence of *H. non-scripta* (MN824434) is 155,035 bp. It comprises the LSC spanning 83,947 bp, the SSC spanning 18,496 bp, and two IR regions, each of which is 26,296 bp in length. The plastome contains 85 protein-coding genes, 38 tRNA genes, and 8 rRNA genes. Of these, seven protein-coding genes, eight tRNA genes, and four rRNA genes are duplicated in the inverted repeats. The protein-coding gene, *infA*, is a pseudogene due to premature stop codons. *Hyacinthoides non-scripta* is sister to *B. japonica*, within the monophyletic Scilloideae subfamily (Figure 1).

**Figure 1.**
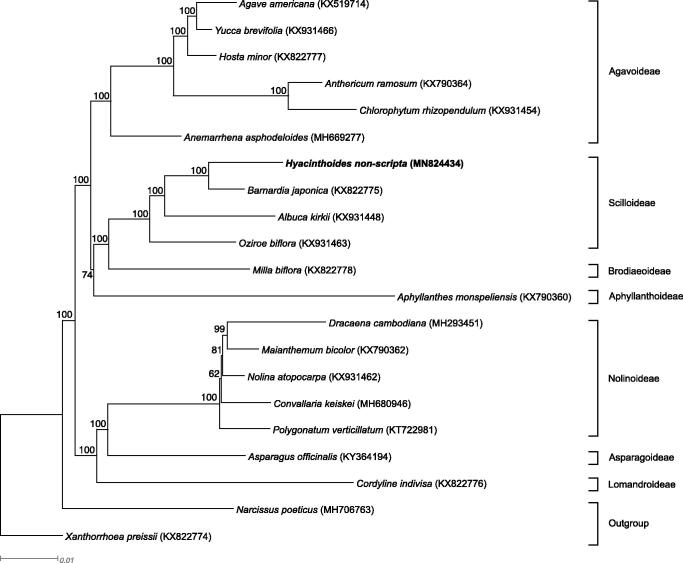
RAxML output tree based on 21 complete plastome sequences. Bootstrap support values are shown at each branch. GenBank accession numbers are given in brackets, subfamilies of the samples are shown on the right. Text in bold shows the plastome developed in this study.
